# From viral assembly to host interaction: AFM’s contributions to virology

**DOI:** 10.1128/jvi.00873-24

**Published:** 2024-12-10

**Authors:** Ankita Ray, Joshua D. Simpson, Irem Demir, Victor G. Gisbert, David B. Gomes, Federico Amadei, David Alsteens

**Affiliations:** 1 Louvain Institute of Biomolecular Science and Technology, Université Catholique de Louvain 195141 , Louvain-la-Neuve, Belgium; 2 WELBIO Department, WEL Research Institute 630407 , Wavre, Belgium; Universiteit Gent, Merelbeke, Belgium

**Keywords:** virus, atomic force microscopy, single-molecule biophysics, force spectroscopy, entry receptors, nanoindentation

## Abstract

Viruses represent a diverse pool of obligate parasites that infect virtually every known organism, as such, their study is incredibly valuable for a range of fields including public health, medicine, agriculture, and ecology, and the development of biomedical technologies. Having evolved over millions of years, each virus has a unique and often complicated biology, that must be characterized on a case-by-case basis, even between strains of the same taxon. Owing to its nanoscale spatial resolution, atomic force microscopy (AFM) represents a powerful tool for exploring virus biology, including structural features, kinetics of binding to host cell ligands, virion self-assembly, and budding behaviors. Through the availability of numerous chemistries and advances in imaging modes, AFM is able to explore the complex web of host-virus interactions and life-cycle at a single virus level, exploring features at the level of individual bonds and molecules. Due to the wide array of techniques developed and data analysis approaches available, AFM can provide information that cannot be furnished by other modalities, especially at a single virus level. Here, we highlight the unique methods and information that can be obtained through the use of AFM, demonstrating both its utility and versatility in the study of viruses. As the technology continues to rapidly evolve, AFM is likely to remain an integral part of research, providing unique and important insight into many aspects of virology.

## INTRODUCTION

Viruses are a class of pathogens that infect virtually all organisms (1, 2) and were first discovered in 1892 by Ivanovski and Beijerinck (3, 4). Viruses have evolved over millions of years and have adapted to their hosts, existing in constant conflict with the immune system of their host, and, thus, have gained the crucial molecular components to evade detection and facilitate their life-cycle (5). Given this evolutionary arms race, there is a constant need to study viral properties and behaviors for emerging pathogens, new strains, and potential zoonoses, necessitating the expansion of techniques and technologies. Characterization of key aspects of viral biology, such as landing, binding, and entry, can provide critical information relevant to the development of novel therapeutics and treatment strategies. However, the process of virus entry into the host cell through receptors is a dynamic and multi-step process, including binding to numerous receptors such as cellular glycans, lipids, and proteins, making these biological systems very difficult to comprehend (6).

Traditionally, virus infection/entry mechanisms are mainly studied using molecular biology, genetic approaches, or imaging techniques such as glycan microarrays, confocal microscopy, and cryo-electron microscopy (cryo-EM) which has led to significant progress in identifying new viral receptors (7–10). However, single-molecule techniques such as atomic force microscopy (AFM) have gained significant attention, improving our understanding of the molecular mechanisms underlying viral attachment to the cell surface, the first step in viral infection.

**Fig 1:**
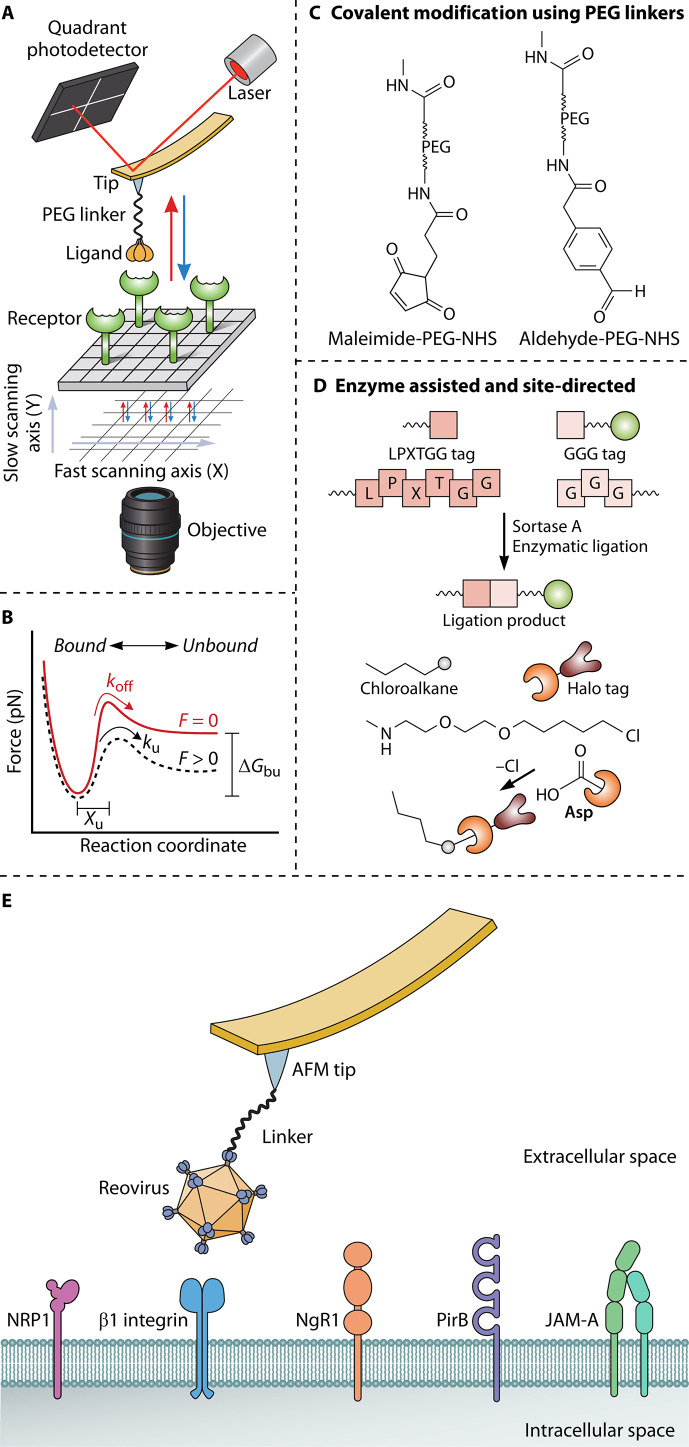
(**A**) Diagrammatic representation of an AFM interfaced with a confocal laser scanning microscope. The functionalized AFM tip is interacting with the receptor-coated model surface, and the force (in pN) is measured by vertical deflection (given in Volts) on the quadrant photodetector; (**B**) free energy landscape of ligand–receptor binding dynamics as given by a two-state Bell–Evans model for single-bond rupture; (**C and D**) cartoon showing functionalization of AFM tips with a distensible PEG linker with specific molecular handles for covalent conjugation of proteins, viruses etc. (top) and enzyme assisted methods for site-specific attachment (bottom); (**E**) a single Reovirus particle tethered to the AFM tip interacting with the plethora of receptors available on the surface of mammalian cells.

The stylus profiler, invented by Gustav Schmaltz in 1929, revolutionized surface measurement by providing a means to record topography. The device worked by moving a surface under a sharp stylus that was mounted on a cantilever. As the stylus moved across the surface, its vertical displacement was tracked, and these movements were monitored by a system that involved reflecting light off a small mirror attached to the cantilever. The reflected light’s motion was recorded onto photographic film, allowing precise measurement of surface features. Additionally, modern (11) AFM technology also builds on the prototype of a scanning tunneling microscope (STM) developed in 1986 by Binnig, Gerber, and Quate (12) and has gone through many technological advancements, resulting in a variety of AFM modes that have aided single-molecule research (Table 1). AFM is a sensitive and label-free technique that allows forces to be measured under close to physiological conditions with unprecedented temporal and spatial resolution. It consists of a sharp tip (nominal radius in the range of a few nanometers) that raster-scans the surface placed on a piezoelectric scanner (Fig. 1A) (12). As the tips move over the sample, it undergoes bending (vertical and torsional) due to a gamut of forces (H-bonding, ionic, π-π, salt-bridge, van der Waals, etc.) acting between the tip and the sample. A direct readout of forces (ranging between a few pN to several nN) is obtained by measuring the vertical laser deflection on a quadrant position-sensitive photodetector. Unlike other microscopes, AFM can also be used in force spectroscopy mode to obtain the nanomechanical information of viscoelastic biological samples (13). Over the years, AFM has already been used in virology research ranging from imaging of viruses to nanoindentation and force spectroscopy (Table 1).

**TABLE 1:** Different AFM modalities that are routinely employed in nano-virology research (14–37)

AFM modalities for viruses	Information	Notes	References
Imaging of viruses	Quantitative and qualitative properties of viruses: virus size, shape, external structures, and surface topography	Measurements can be performed both in dried and hydrated states which allow to estimate the degree of shrinkage they undergo because of dehydration	(15–17)
	Imaging the distribution of virus particles on the cell surface	Virus-infected cells could be visualized	(19, 20)
Dynamic force spectroscopy	Kinetics and thermodynamics of bond formation	Quantify the energy landscape of receptor ligand interaction	(14, 37)
Single virus force spectroscopy	Receptor localization and mapping	Enables for binding side localization directly on live cells and structure of cells to be linked with receptor distribution	(29–32)
	Binding frequency and bond strength	Binding frequency increases with contact time. Fitting the data using a pseudo first- order kinetics allows to extract the kinetic association rate (*k*_on_) of the interactions	(33–36)
Force spectroscopy-based virus nanoindentation	Mechanical properties of virions	Viral mechanics are governed by the stiffness of the capsid shell, frequently in conjunction with the internal pressure from the tightly packed genome	(21–23)
	Deformability (stiffness, *k*), elastic modulus (Young’s modulus, *E*), the energy required for mechanical failure (toughness, *T*), and the limits to fatigue for virions	Spring constants and *E* of different viruses ranges between 0.018 N/m to 1.3 N/m and 0.14 GPa to 2.8 GPa, respectively. This could be attributable to change in capsid size, thickness, and geometry as well as inter- and intrasubunit molecular interactions	(18, 24–28)

Understanding the behavior of molecules under non-equilibrium conditions involves concepts from non-equilibrium thermodynamics, which includes the study of how external parameters (force, temperature etc.) drive systems away from equilibrium and how these parameters influence molecular interactions and bond resilience. By pulling on a bond under increasing pulling speeds, bond strengths are measured under out-of-equilibrium conditions that favor bond rupture by lowering the activation energy for dissociation. Single-molecule force spectroscopy (SMFS) and dynamic force spectroscopy (DFS) methodologies are routinely used to measure kinetic on- and off-rates (*k*_on_ and *k*_off_) of virus-receptor interactions and are assessed by DFS data fitted with appropriate models (14). The three models that are routinely employed to describe this process are the Bell-Evans (38), Dudko-Hummer-Szabo (DHS) (39), and the Friddle models (40). The classical Bell-Evans (BE) phenomenological model predicts a linear dependence of rupture forces on the natural logarithm of the loading rate and gives access to the intrinsic *k*_off_ and distance to the transition state (*x*_b,_ expressed in nm) (Fig. 1B) Bonds break when enough force is applied to overcome the activation energy barrier that stabilizes the bond. The height and shape of this barrier determine the propensity of the bond to break under external force. This is one of the classical models used to describe bond rupture under external force. Simply put, the BE model computes the relationship between the dissociation rate of a bond and the loading rate in dissociation pathways that involve one transition state. From the viewpoint of a simple reaction coordinate with one transition state, *x*_b_ is the distance along the reaction coordinate from the minimum energy configuration (bonded state) to the top of the energy barrier (transition state) (Fig. 1B). This distance is directly related to the loading rate (rate at which force is applied) and the bond rupture force. In principle, higher forces are required to rupture bonds with shorter *x*_b_ values, as the bond is more resilient to the applied force. The force required to rupture the bond (mean rupture force) depends on the loading rate (how fast bonds are pulled) and the height and width of the energy barrier. The main limitation of this model is that it fails to account for the multidimensional energy landscapes or complex systems. Hence, to circumvent this limitation, the BE model was further developed by the DHS model, which accounts for the shape of the free-energy surface, and factors in the dependence of *x*_b_ on the applied force. The DHS model allows for the differentiation between different energy barrier shapes (parabolic, cusp-like, etc.) and is more suitable for bonds that exhibit complex force-dependent behavior with multiple transition states (41, 42). By invoking the Friddle model, where rebinding at low-pulling rates is considered, the height of the activation energy barrier (Δ*G*^‡^) can be readily obtained. It improves on earlier models by incorporating the effect of thermal fluctuations on bond stability. These are crucial to describe the interface mechanostability and dynamics of the biomolecular complex and are intrinsically related to the bond lifetime.Such biophysical investigations offer insights into the binding affinities of viruses and can discriminate between closely related mutants that differ by a few amino acid residues.

The versatility of AFM lies in its ability to function as a nanoscopic chemical probe. Chemical functionalization of AFM tips is a crucial step for specific molecular recognition, force measurements, and imaging applications (43, 44). Cantilevers can be readily functionalized with a plethora of molecular constructs including but not limited to purified proteins, cells, and viruses allowing the tip to function as a molecular sensor that can gauge interactions between bio-samples and their environment using SMFS approaches. Polyethylene glycol (PEG) linkers, particularly heterobifunctional ones, play a crucial role in AFM to detect specific molecular interactions. Their flexibility, hydrophilicity, and ability to reduce non-specific interactions make them ideal for applications in SMFS (45). Long and distensible heterobifunctional PEG linkers are attached to the AFM tip via one of their molecular handles involving straightforward reactions for instance between NHS-ester (N-hydroxysuccinimide) and amine or maleimide and thiols (Fig. 1C). While covalent attachment via amide bond formation using EDC (1-ethyl-3-(3-dimethylaminopropyl)carbodiimide) and NHS or aldehyde-PEG-NHS linkers is a facile technique to graft molecules, a more controlled geometry of grafting can be obtained by enzyme-mediated approaches (Sortase-mediated coupling, HaloTag, SpyCatcher, etc.) (46–48) that offer precise and robust ways to modify AFM tips for targeted interactions (Fig. 1D).

Albeit, the possibility of functionalizing tips with a host of protein (49) or organic molecules (50, 51) continues to expand, there are certain limitations that deter progress made in this field. In particular, such techniques involve multiple steps, including amino-functionalization with silanes (52), choice of right linkers with orthogonal functional groups while ensuring proper orientation of molecules (53). Moreover, the sensitivity of certain biological molecules to environmental factors (e.g., pH, temperature) adds to this complexity. Inconsistent tip and surface functionalization strategies lead to irreproducible data sets which are extremely non-trivial to analyze. This lack of reproducibility is of significant problem in quantitative studies, such as force spectroscopy, where repeatability is crucial for statistical validity. Additionally, even after successful grafting, AFM tips can still suffer from non-specific interactions between the tip and the sample, particularly in biological experiments involving a milieu of complex receptors. To ensure the specificity of probed interactions, several blocking or control experiments should be conducted that can differentiate between specific and non-specific interactions. Hence, careful and rigorous considerations should be made to validate the specificity of interactions while limiting the occurrence of user induced or experimental aberrations.

**Fig 2:**
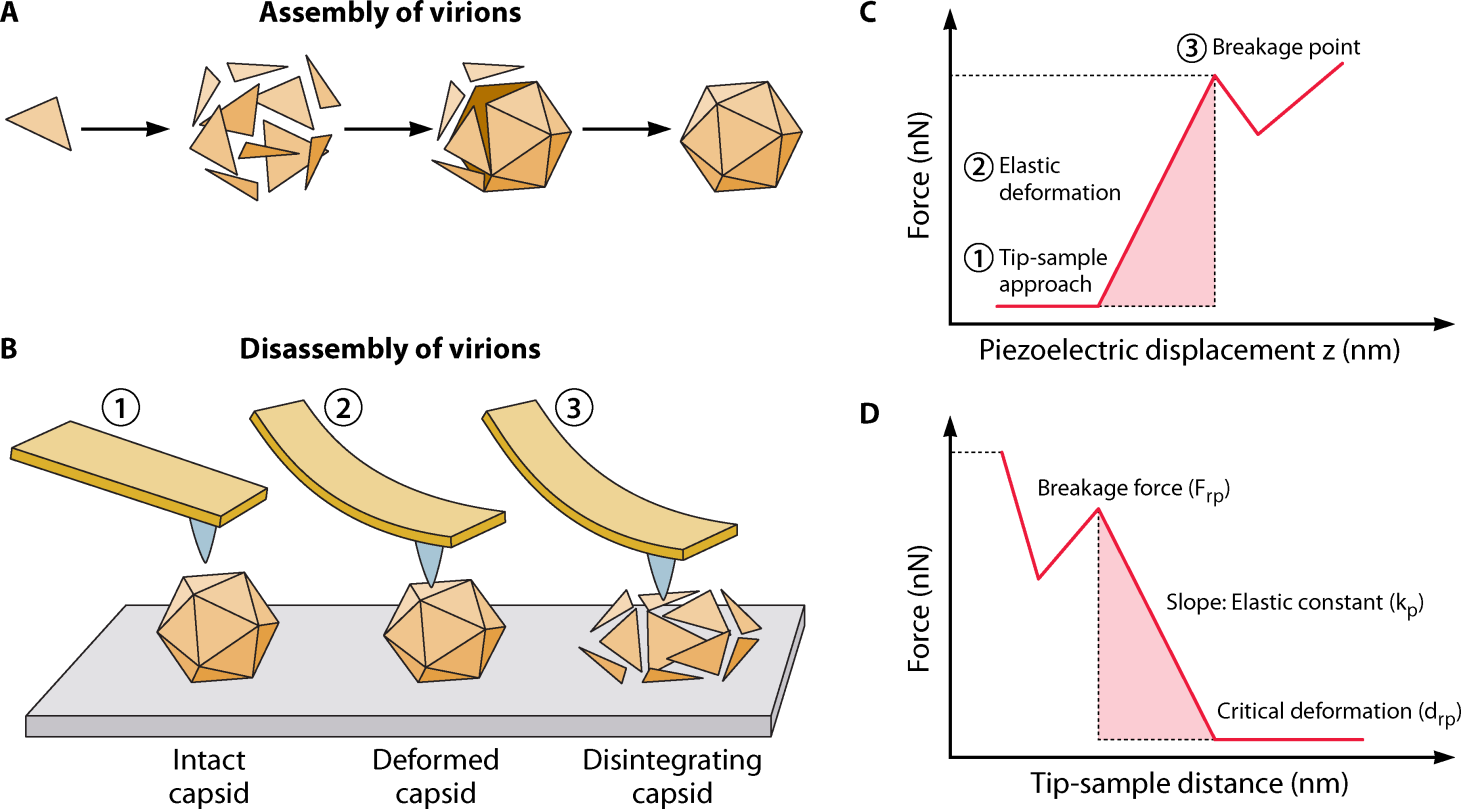
(**A**) Cartoon representing the natural assembly of viral particles by self-assembly of the capsids; (**B**) AFM-based nanoindentation of single viral particles in three consecutive steps: the tip is positioned far from the virion (1), the tip is slowly brought into contact and force is applied on the viral particle (2), force is increased until deformation of the virion is observed leading to breakdown of the lattice and concomitant loss of structural integrity (3). (**C and D**) Mechanical properties of virus particles determined by AFM-based indentation experiments as described in panel B.

Viruses can be imaged by AFM to study their structures and fundamental biological processes at high resolution in liquid (15, 54, 55). AFM images can measure fundamental parameters of virus particles such as their architecture, diameter, length, or spherical/helical shapes (16). Different AFM modes, such as contact mode and oscillation mode, can be employed to image virus particles. In contact mode, the tip directly engages with sample and scans the sample surface while keeping the force constant. This imaging mode provides high-resolution images; however, constant force applied during scan may deform soft samples. On the other hand, in oscillation mode, the tip oscillates near its resonance frequency while scanning the sample surface and the variations in the oscillation amplitude show the surface topography (56). In this mode, the tip is not in contact with the sample surface which significantly reduces lateral forces exerted by the tip. Moreover, besides imaging, AFM can be used to measure mechanical properties of virus particles such as stiffness (*k*), elasticity/Young’s modulus (*E*), toughness (*T*), and resistance to mechanical failure through nanoindentation allowing the structure of virus particles to be linked with their biophysical properties (17, 18, 57, 58). (Fig. 2) For nanoindentation measurements, force-distance (F-D) curves are converted into force-indentation (F-I) curves by subtracting the deflection of the cantilever. This way, stiffness (*k* expressed in N/m) and intrinsic Young’s modulus (*E* expressed in Pa) of virus particles can be extracted and quantified by using appropriate models (i.e., Hooke’s law and Hertz model). Such nanomechanical measurements as well as surface imaging provide significant information on the capsid shell of the virus which represents its interface with the surrounding.

The other use of AFM in virology includes the understanding of viral entry process model surfaces as well as directly on live cells and is not restricted to imaging or probing mechanical features of viruses. The development of high-speed AFM (HS-AFM) opens up possibilities to observe the dynamic behavior of biomolecules such as proteins at high spatiotemporal resolution (59, 60). This enables to investigate virus-cell interactions at a high temporal resolution as well as conformational changes of viral proteins (such as membrane glycoproteins) that were previously not possible with conventional AFM.

## IDENTIFICATION OF VIRAL ENTRY RECEPTORS AND PATHWAYS

Critical to the viral lifecycle, the early stages of cellular infection include landing, binding, and entry. Upon encountering a potential host cell, a virus needs to adhere to the cell surface, successfully navigate to a suitable entry mechanism, and subsequently initiate the required mechanism. To achieve this, many viruses first bind to low-affinity attachment factors or co-receptors (61), which then serve to anchor the virus to the cell, but with enough freedom to allow the virus to maneuver until it encounters a suitable receptor, enzyme, or other mechanism that facilitates its entry into the cellular milieu (61). Due to the high sensitivity afforded, AFM is an excellent tool for the study of these processes (44), especially when combined with other imaging modalities, such as confocal microscopy. Among the key applications of AFM, single virus force spectroscopy (SVFS) and DFS (Table 1) can garner useful information regarding the localization, kinetics, and dynamics of these interactions (61). Pertinent to understanding the underlying mechanics of cellular infection and providing valuable insight for the design of inhibitory therapeutics, AFM is often used to explore the thermodynamics and kinetics of key receptor interactions, for instance, in the case of SARS-CoV-2.

AFM was used to resolve the forces between the SARS-CoV-2 spike protein and its primary receptor, angiotensin-converting enzyme 2 (ACE2) covalently anchored on model surfaces and extrapolated to physiologically relevant conditions on living A549 cells. Using functionalized cantilevers as chemical nanoprobes, studies of the RBD^WT^/ACE2 interface showed that the spike/ACE2 interaction is not only strong (*K*_D_ ~120 nM) but also involves multiple binding sites, which contribute to the overall stability of the virus attachment (36, 62). By varying the time [range of milli-second (ms)] that the tip interacts with the surface, binding frequencies (BFs; percentage of specific adhesion events) can be obtained which are approximated with pseudo-first kinetics to provide the *k*_on_ values (units: M^−1^ s^−1^). However, to obtain *k*_off_ values (units: s^−1^), DFS experiments are performed by sequentially increasing the loading rate [obtained by multiplying the cantilever spring constant (N/m) with retraction velocities (nm/s)]. This helps probe the strength of molecular bonds much beyond the thermally activated regime and provides direct access to the energy landscape under out-of-equilibrium conditions (39, 63).

After a period of brief evolutionary stasis, there has been an upheaval in the number of circulating variants of concern (VoCs) of SARS-CoV2 with a wide range of antigenicity and disease severity. These VoCs (Alpha, Beta, Omicron, Delta, etc.) harbor several key mutations at the receptor-binding domain (RBD) that have serious implications for the virus’ transmissibility, pathogenesis, and ability to evade immunity (34). Understanding the single-molecular mechanism employed by the virus to recognize and attach strongly to cognate receptors is a crucial area of research in the fight against the COVID-19 pandemic. In this context, by a combination of AFM and steered molecular dynamics (SMD), the mechanical forces were probed with high spatial resolution under far from equilibrium conditions. While AFM enables the observation of interaction between individual molecules in real time, SMD provides valuable insights into the binding mechanism under extremely high loading rates (34). In addition, we leveraged the power of AFM to study the blocking potential of antibodies derived from convalescent patient sera. Because AFM can help discriminate forces down to the pN level, it was able to provide remarkable insight into different binding affinities exhibited by the new variants of concern (VoCs). More recently, this technique has been used to resolve the forces acting at the virus/cell interface in the presence of monoclonal antibodies and patient sera with the newer circulating VoCs offering a new method to investigate the neutralization breadth of antibodies (34).

In a similar vein, AFM studies have shown that the DC-SIGN and L-SIGN receptors may function as additional receptors involved in the recognition of viral glycoproteins of SARS-CoV-2 (64). These two transmembrane receptors share a carbohydrate recognition domain (CRD) with nearly 80% sequence homology and bind to the spike protein through its glycosylated regions (65, 66). Both DC-SIGN and L-SIGN recognize and bind to the SARS-CoV-2 spike protein, but they may do so with different affinities and specificities due to variations in their CRDs causing differing glycan preferences (66), as demonstrated by AFM on model surfaces and living cells (64). Through SMFS experiments between DC-, L-SIGN model surfaces and spike protein tethered on the AFM tip, better binding kinetics were observed in the case of the L-SIGN receptor (*K*_D_ ~20 nM for L-SIGN/S1 vs ~420 nM for DC-SIGN/S1) (64). The significantly different binding behaviors indicated that although L-SIGN may have some role as an alternative entry receptor, both may function as routes to aid infectivity through processes such as transinfection, or binding of SARS-CoV-2 to the cell surfaces (64). The specific glycan structures on the spike protein recognized by these receptors may influence how viruses first *land and then seek* other available receptors, such as the ACE2 receptor, to establish a tighter binding (64). Through characterization of host-virus interactions by AFM, it is clear that such behaviors are common across many virus phylogenies.

Another example that illustrates the utility of AFM in deciphering the complex web of interactions required for viral infection is its use in the study of mammalian orthoreovirus (reovirus) (67). Despite clinical translation as an oncolytic cancer therapeutic (68) and a suspected role in the pathogenesis of celiac disease (69, 70), reovirus represents an important area of research because many of its binding partners remain enigmatic. Reovirus has been demonstrated to possess a wide array of ligand interactions mediated by the outer layer of its concentric capsid protein shells (71). These capsid-ligand interactions have been identified by AFM to function both individually and in concert to engage an array of mammalian surface molecules (Fig. 1E). At current, reovirus is known to bind Junctional Adhesion Molecule-A [JAM-A (72, 73)], β_1_ integrins (74), Paired Immunoglobulin-like Receptor B [PirB (75)], neuropilin-1 [NRP1 (67)], Nogo Receptor 1 [NgR1 (76)], and surface glycans (37). Many of these interactions occur via the more exposed σ−1, σ−3, or λ2 capsid proteins although NgR1 and NRP1 were identified to engage higher order structures, requiring multivalent bonds with heterooligomer assemblies, as has been confirmed using SMFS (67, 76). It remains to be seen whether the interactions identified with many novel reovirus receptors are ancillary to JAM-A, predominantly serve as unique alternative entry pathways, or function synergistically as co-receptors. To shed light on this, AFM can be utilized in conjunction with confocal microscopy to monitor recruitment of endocytic machinery in response to receptor engagement, as has been performed for β_1_ integrins (74), typically this information is garnered through ensemble infectivity studies.

In addition to providing an important role as attachment factors, the impact of surface glycans has been identified as a crucial for reovirus infection, with different serotypes preferring specific glycans, for instance, the Dearing strain (T3D) engages sialic acids (specifically α-linked 5-*N*-acetyl neuraminic acid) (77), whereas the Lang variant (T1L) binds ganglioside GM2 (78). Through AFM, it has been demonstrated that these surface glycans also operate as co-receptors, inducing conformational changes in the reovirus σ−1 protein, which consequently improves its affinity for JAM-A, improving both binding probability and kinetics on model surfaces and living cells (37, 79). The mechanics of the extended conformer have also been explored using mutations that impact the rigidity of the reovirus σ−1 protein homotrimer, wherein DFS could distinguish that more rigid complexes formed by crosslinking the head domain of the protein complex improved affinity for JAM-A on living cells despite reducing the number of multivalent bonds involved (35, 79). In the case of the T1L serotype, the improvement in the dissociation constant (*K*_D_) extracted for mutants cross-linked in the σ−1 head domain was almost 10-fold lower than the wild-type virus (35). This improvement in specificity at the expense of the ability to traverse the cell surface via exploitation of lower affinity bonds was also confirmed through single particle tracking, wherein it was found that once bound on the surface of JAM-A positive cells, the mutant would laterally diffuse less than its counterparts (35).

## ASSEMBLY OF VIRAL PARTICLES

Virus formation begins with the self-assembly of the capsid proteins. During the capsid formation, hundreds of identical capsid proteins assemble driven by electrostatic (80), protein-protein, and genome-protein interactions. Most viruses assemble into an empty capsid into which the genome is later inserted by means of a rotating molecular motor. In other viruses, such as ssRNA viruses, the capsid assembles around the nucleic acid. For example, in the tobacco mosaic virus (80, 81) (TMV), the capsid molecules self-assemble around the RNA molecule forming a cylindrical structure. Under physiological conditions, protein clusters are formed, but the presence of the RNA molecule is required for the full assembly of the capsid During virus self-assembly, three different stages have been observed: lag phase, nucleation, growth, and saturation (82). As the concentration of monomers increases, they bind to form small protein groups (oligomers) and the subunits of the capsid lattice (Fig. 2A). In some cases, chemical factors or external activation are required to activate the assembly. Most techniques study the self-assembly of viruses using techniques that measure the evolution at the bulk level, unable to measure intermediate steps or the evolution of individual virions. However, AFM enables *in situ* observation at the molecular level of the assembly process. For example, the self-assembly process of the human immunodeficiency virus (HIV) capsid protein lattice has been observed at the single-molecular level by using high-speed AFM (83) under quasi-physiological conditions. Initially, after immobilization on the substrate surface, oligomers are formed (lag phase) until the surface is covered by at least 50% of oligomers, triggering the formation of nucleation points. The formation of these points was only observed when a minimum density of oligomers was detected on the surface, and the density reached supersaturation. During the growth phase, it was observed that neighboring patches with similar orientations were able to fuse cooperatively. The assembly of the hexamer is a stochastically reversible process with different pathways of assembly and disassembly. After the hexamer is fully formed, the molecule remains stable and the lattice growth continues in neighboring areas.

High-speed AFM also enables the study of the nonlytic virus egress post-assembly (84, 85). By imaging the newly formed viruses passing through the membrane phase (86, 87), it was observed that three different stages dominate the budding mechanism. During the early stages of virus budding, the virus deforms the cellular surface, and a protrusion with a height of 40–70 nm of is observed. Afterward, the virus remains at a stationary phase during which no change in the height or geometry is observed. At the final stage, the virus is either released or reabsorbed by the membrane. It was observed that the length of the stationary phase, the speed of the budding, and the efficiency of the virus release depend on the height of the virus over the surface, suggesting the presence of various distinctive pathways for virus budding.

Once the capsid has been assembled, some viruses will undergo a maturation process that stabilizes the capsid. The maturation consists of a series of irreversible reactions that convert the structure of the capsid into a mature (infectious) virion. These reactions stabilize the structure of the capsid. In dsDNA viruses after maturation, the pressure of the capsid increases up to 30 atm which increases the mechanical resistance of the virus and facilitates the injection of the viral genome into the host (88). The elastic properties and thickness of the capsid are also affected by maturation.

## STIFFNESS OF VIRAL PARTICLES

Usually, the structure and behavior of the viruses are measured using techniques that measure the behavior of a large number of virions. However, biological processes are highly uncoordinated, with the presence of multiple intermediate events that are hidden when observing the ensemble properties. For this reason, techniques such as the AFM, which can resolve the structure and mechanics of single virions, are very useful for the understanding of the virus at the molecular level.

The AFM enables high-resolution measurement of the structure of the virus (15, 54, 55) *in situ*. This application enabled the characterization of the coat proteins of individual virions, measurement of the structure and height of the Moloney murine leukemia virus before, and after attaching to the host cell showing the clustering of the virus before infection, the icosahedral symmetry of the Ty3 retrotransposon, or the protein distribution in the capsid of the Mason-Pfizer monkey virus (15). AFM also enabled the measurement of the structure and uncoating of very soft virions such as HIV-1 (89), human picobirnavirus-like particles (54), or adenovirus (55).

In addition, the AFM can apply controlled forces over very small areas which enable characterization of the mechanics and internal structure of the virus (Fig. 2B). The interplay between biomechanics and biological function has been demonstrated in certain viruses. It has been shown that stiffness or viruses change as an effect of maturation. For example, the human adenovirus (HAdV), murine leukemia virus (MLV), and human immunodeficiency virus (HIV) show changes in stiffness during their maturation phases, enabling efficient entry into and exit from host cells (88, 90, 91). Furthermore, mutations in the coat protein Minute virus in Mice impair its infectivity due to changes in the stiffness of the capsid (92). However, for the human adenovirus of type 5, mutations in the viral capsid double the stiffness of the virus and show no effect in the virus infectivity (93). Using F-I curves (94), the stiffness (*k*) and Young’s modulus (*E*) of the virus are measured. The Young’s modulus is an intrinsic property of the internal structure of the virus which depends on the internal pressure of the genome, state of maturation, the presence of membrane proteins (95), and local properties of the capsid (Fig. 2C and D). On the other hand, the stiffness also depends on the size and geometry of the particle. For this reason, the value of *E* provides a more meaningful description of the biological properties of the virus although the complexity and multilayer nature of the capsid makes the measurement of this property difficult. Typically, the virus is modeled using a thin shell model. In that case, the relation between the stiffness and the Young’s modulus is given by *k = Eh*^2^*/R,* where *h* is the thickness of the shell and *R* is the radius of the virus shell (25, 96, 97). The values of stiffness and elasticity greatly vary between the different species of viruses. The stiffness of SARS-CoV-2 was found to be 0.013 N/m (98), while the influenza virion presents a stiffness of 0.05 N/m (95). In comparison, the stiffness of the tobacco mosaic virus is 0.8 N/m with a Young’s modulus of 920 MPa (99).

Another parameter observed during the mechanical measurement with nanoindentation is the breaking force. The breaking force indicates the maximum force the virus can withstand before showing irreversible damage or collapsing (100). Typically, the breaking force is in the order of a few nN, and a maximum indentation of ~10% (101). Since these deformations compromise the structure of the virus, it is recommended to perform the AFM measurements with forces below the critical limit. However, it has been reported that repeated measurement of the virus within the elastic limit can still produce fatigue in the virus leading to mechanical disintegration. Under these conditions, repeated imaging with the AFM of the virus could be used to measure the different stages of disassembly (102) and aging (55). As the capsid gradually opens, the genome is exposed to the environment (103). Similarly, the measurement of the surface of the vaccinia virus during enzymatic digestion enabled the dissection of the insides of the virus at different stages of the virus disassembly (15).

The maturation state of the virus also affects the Young’s modulus and the stiffness. In the case of dsDNA bacteriophages, the virus increases the strength of its shell after maturation (104). In the Bacteriophage HK97, the stiffness increases from 0.018 N/m to 0.12 N/m after cleaving the Δ-domain of the gp5 capsid protein (28). In contrast, the HIV-1 virus undergoes a dramatic softening after maturation going from 3.2 N/m to 0.2 N/m (91). During this process, the HIV particles decrease the thickness of the protein shell, which increases the infectivity of the virion.

Finally, during infection of the host cell, the virus undergoes another change in the stiffness to induce capsid disassembly. Inside the cell, the reverse transcriptase transforms the compressed ssRNA inside the HIV-1 virus into ssDNA filaments. This induces the formation of strong filaments that produce an increase in the internal pressure of the core. It has been observed with AFM that during this process the stiffness of the virion increases by a factor of 3. This increase in the internal pressure eventually breaks the membrane, producing capsid disassembly (105).

The mechanical and chemical stability of the virus capsid makes it ideal for the delivery of not only the virus genome but also other fragile cargo such as proteins, molecules, or nanoparticles. The transport of these particles inside the virus capsid allows the protection and controlled delivery of the cargo, while the controlled self-assembly of the virus-like particles is easy to produce and modify. For this reason, the fabrication of virus-like particles has raised interest in nanotechnology and medical applications. To study the stability of this artificial capsid, the study of the stability of the particle and the interplay between the cargo and the capsid is crucial. For example, the capsid of the *Salmonella typhimurium* bacteriophage P22 could be filled with a cargo consisting of EGFP or β-glucosidase from the hyperthermophile Pyrococcus furiosus (101). The size of the capsid increased between 10% and 20% after inserting the cargo, which also increased the stiffness of the particle from 0.21 N/m to 0.27 N/m due to an increase in the internal pressure produced by the cargo. However, the maximum indentation the capsid can withstand before breaking remained constant, showing the importance of the resistance of the capsid.

## SUMMARY AND FUTURE PERSPECTIVES

AFM is an indispensable tool in nanoscale research, offering unparalleled resolution and the ability to probe various physical properties (106). In the field of virology, AFM can be a very useful tool to improve our understanding of the biophysical interactions between viruses and hosts. This technique offers significant advantages for the study of viruses, allowing for high-resolution imaging, measurements of mechanical properties, and insights into virus-cell interactions. In this review, we have traced the evolution of this technology and how it has challenged the study of viruses by offering unique capabilities that complement traditional virology techniques like cryo-EM or infectivity assays. A great deal of focus was devoted to showcase some key applications of AFM to study viruses and their properties. Spanning from high-resolution imaging, with detailed topographical images of viral particles at the nanometer resolution, to the intrinsic biophysical properties of virus, such as their mechanical properties, AFM seems to be crucial to deepen our knowledge in viral assembly, morphology, and interactions with host cells. Moreover, these measurements provide new insights into the stability and infectivity of viruses, as well as their resistance to environmental stresses and antiviral agents (107). Following the development of single-molecule spectroscopy, AFM can be used to study the interactions between viruses and host cells at the nanoscale, allowing the exploration of energy landscapes of primary and ancillary cellular receptors with viral ligands to extract the ligand-receptor kinetic parameters. Furthermore, by imaging the binding and entry of viruses into host cells, we have shown how AFM can effectively describe the viral entry pathway into the host cell, which, in turn, can be useful to confirm potential targets for antiviral therapies. Lastly, due to the versatility of this technique, AFM also enables the manipulation of individual viral particles, hence allowing a more comprehensive understanding of viral assembly, disassembly, and the effects of antiviral drugs on virus structure and function under different physiological conditions.

Despite the invaluable potential AFM brings into virology, there are still challenges and technical limitations that must be considered and tackled. First, viral samples preparation for AFM imaging can be challenging, as it requires maintaining the native structure and functionality of viruses. Immobilizing viral particles without altering their properties is essential for accurate and reproducible AFM measurements. Additionally, it is crucial to minimize the interaction between the AFM tip and the viral sample, which can bias the measurements by introducing a variable tip-sample contact area. Minimizing tip-induced artifacts and ensuring reproducibility of measurements are critical for trustworthy data. Additionally, it is rather important to ensure the stability of any external environmental factor, as temperature, humidity, and/or vibrations can affect AFM measurements. Developing robust environmental control systems can be time-consuming but are essential for consistent and accurate AFM readouts. Concurrently, interpreting AFM data, particularly mechanical property measurements, can be a difficult time-consuming task, usually requiring a trained expert. Advanced theoretical and automated approaches to analyze DFS data and computational models in combination with analytical techniques are needed to accurately correlate AFM measurements with the biological properties of viruses. Hence, as data set size increases, the use of advanced artificial intelligence (AI) computational techniques such as deep and machine learning to analyze AFM data has been drawing increasing attention from the scientific community (108–110). The latter can reduce significantly the time effort of analyzing thousands of force-distance curves generated in a single experiment once a robust training set has been provided to the algorithm.

Taken together, the literature summarized in this review demonstrates the impact that AFM has already had in virology. However, it seems that we are still scratching the surface of the full potential of this technique to better understand the various aspects of viral infection mechanisms. Driven by the advancements in AFM hardware and software, and cross disciplinary approaches, include achieving real-time dynamics imaging via high-speed AFM (HS-AFM) enabling the real-time imaging of dynamic processes involving viruses, such as assembly, disassembly, and interaction with host cells. This will provide unprecedented insights into the life cycle of viruses and the effects of antiviral agents. To gain further insight into viral processes at multiple scales, the integration of AFM with other microscopy techniques, such as confocal microscopy and cryo-EM, will play a fundamental role allowing researchers to correlate topographic and mechanical data. The investigation of this correlation can be strengthened by interfacing specialized multifrequency AFM mode (111) with infrared spectroscopy (IR) to provide the nanomechanical and chemical properties of viruses (112). This knowledge can be applied to design more effective antiviral strategies and improve vaccine formulations.

Furthermore, by functionalizing AFM tips with antibodies or aptamers, researchers can create nanoscale sensors for early and accurate detection of viral infections (113). New antiviral strategies and improved formulations will be supported by advanced single-molecular studies, which enable detailed investigations of viral/protein interactions, conformational changes, and the effects of mutations on viral fitness. Lastly, AFM can become a key player in the field of biosensing and diagnostics, wherein it can be interfaced with other biosensing techniques to develop sensitive and specific diagnostic tools for viral detection (114).
